# Average current mode controller for bridgeless PFC SEPIC converter with second-order model reduction operated in continuous conduction mode

**DOI:** 10.1371/journal.pone.0291873

**Published:** 2023-10-17

**Authors:** Nor Akmal Rai, Mohd Junaidi Abdul Aziz, Mohd Rodhi Sahid, Abdul Rashid Husain, Waqas Anjum, Wen Yao Low

**Affiliations:** 1 Faculty of Electrical Engineering, Universiti Teknologi Malaysia, UTM Johor Bahru, Johor, Malaysia; 2 Department of Electronic Engineering, Faculty of Engineering, The Islamia University of Bahawalpur, Bahawalpur, Pakistan; Ghani Khan Choudhury Institute of Engineering and Technology, INDIA

## Abstract

This paper proposes an average current mode controller (ACMC) for a single-phase bridgeless power factor correction (PFC) circuit using a single ended primary inductor converter (SEPIC) via second-order model reduction. The superiority of the proposed controller is PFC accomplished at power up to 350 W with high efficiency via the second-order model reduction. The design and implementation of ACMC on the converter operated with continuous conduction mode (CCM) is explained in detail. ACMC forces input current to follow sinusoidal current reference at different power levels and sustain high power factor (PF). The proposed controller is designed based on the theoretical analysis operation of the circuit. For verification, MATLAB/Simulink simulations are carried out and validation through an experiment test rig for 110—220 *V*_*rms*_ input, 100 *V*_*dc*_/ 350 W output prototype at 20 kHz switching frequency. It is proven that the proposed controller strategy accomplishes high PF, high efficiency and conformity with the simulation.

## Introduction

The rapid development of technology using a variety of electronic devices has affected the power quality of the ac system. The losses from these electronic devices have been causing pollution in the electrical system resulting from low power factor (PF). Low power factor causes high peak input current at supply and causes high losses, which provide harmonic distortion to the line system. Hence, power factor correction (PFC) becomes necessary to eradicate losses in all switching power supplies.

PFC sustains a nearly unity power factor by forcing the alternating current input to be in phase with the input voltage. Ac-dc converter normally used bridge rectifier and dc-dc converter to regulate dc output. However, the bridge rectifier contributes to conduction losses to the circuit during operation. Three semiconductor elements are conducted simultaneously, causing an increase of forward voltage drop in the circuit.

The bridgeless converter has been introduced since 1983 by replacing two adjacent diodes in the conventional bridge rectifier with a single switch [1]. This results in eliminating high conduction loss at the input side of the ac-dc converter. The discovery of the converter enhanced to various newer topologies of the bridgeless converter is rolled out with different structures of improvement from the previous bridge converter [2–4]. Numerous studies reported that by eliminating the bridge diode, converter efficiency increases and significantly losses [3–5]. Reduction of cost, high efficiency, weight and maintaining near unity power factor are several advantages of the bridgeless converter. The bridgeless SEPIC converter is one of the eminent converters due to its benefit compared to other dc-dc converters. This converter emits no polarity reversal of output voltage and provides an extensive range of dc output based on the duty cycle. The SEPIC converter is a 4th-order converter; due to having four storage elements in the converter topology, it is not widely investigated due to the controller’s design complexity.

**Fig 1:**
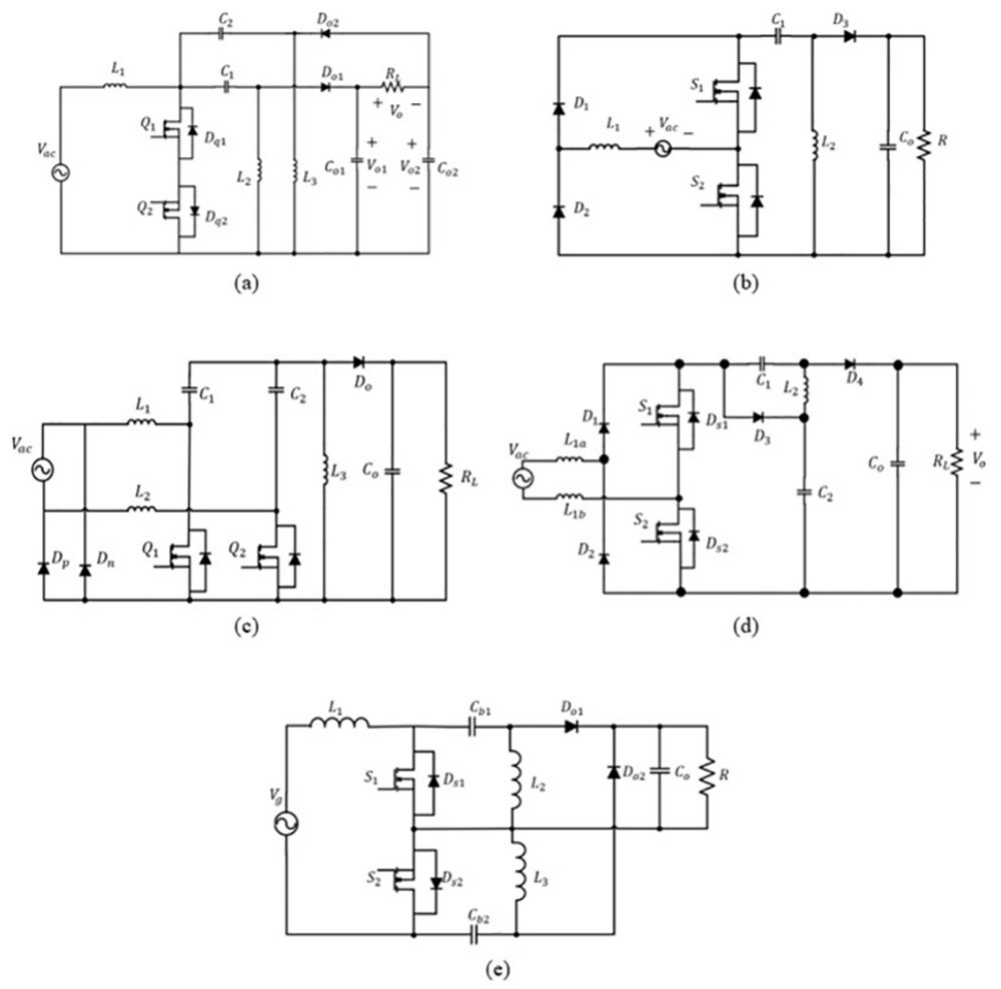
Bridgeless SEPIC converter topologies (a) [6], (b) [7], (c) [8], (d) [9], (e) [10].

The converter has become a hot topic over the last few decades to overcome traditional bridge SEPIC converter front-end PFC efficiency. Five new bridgeless SEPIC topologies have been introduced with different positioning structures of two active switches and elements, as shown in Fig 1.

In [6], the proposed topology combines two SEPICs in one circuit. The first SEPIC is for the positive input source, while the other is for the negative input source. However, the output ripple of this topology is double that of the conventional SEPIC converter due to existing of the two output capacitors. As illustrated in Fig 1(b), [7] proposed switches replace two diodes of the input bridge rectifier of the conventional SEPIC converter. No additional element is used in this bridgeless SEPIC. Since only one diode is used in the current path during each switching cycle, the circuit has a low conduction loss. This topology gives advantages on the simplicity of design, improved power factor, and semiconductor devices offer low voltage stress.

Low conduction losses are achieved by the proposed bridgeless SEPIC converter in [8], as shown in Fig 1(c). This topology is advantageous concerning the continuous connection between input line voltage and output ground through two slow recovery diodes namely *D*_*p*_ and *D*_*n*_ which is equivalent to the traditional SEPIC. This leads to the elimination of high common-mode EMI noise issued in other bridgeless SEPIC converter, additionally the power switch is driven by similar PWM control which makes it easy to design a control circuit. An additional inductor in the input voltage is designed to improve the thermal performance of the topologies. However, this will contribute to a bulky size converter and simultaneously increase the cost.

The efficiency of the bridgeless PFC SEPIC converter proposed in [9] with reduced voltage stress is shown in Fig 1(d). An auxiliary circuit consisting of a diode and a capacitor is used throughout the topology to decrease the voltage stress on the main switch and diode. Due to the voltage stress of the main switch has been decreased to half, the low voltage element can be used. Hence, the efficiency of the converter is enhanced due to low on-state conduction loss, low *R*_*ds*_(*on*) in low voltage rating switch and less forward voltage drop in low voltage rating diode. However, the circuit provides a high number of elements with 4 diodes in the circuit design.

The bridgeless SEPIC converter, as proposed in [10], upgraded several drawbacks in [6] whereby the output resistor is not floating, and only one output capacitor is required, as shown in Fig 1(e). As compared to other bridgeless SEPIC converters, the number of elements that conduct during each cycle is minimise which further reduces conduction losses of the bridgeless converter. The bridgeless SEPIC converters act as a simple dc-dc SEPIC converter at each half cycle.

All the above-mentioned bridgeless SEPIC converter topologies are operated in DCM; hence voltage control technique was employed. It is widely known that the bridgeless converter is capable of working in both continuous current mode (CCM) and discontinuous conduction mode (DCM), relying on its implementation. The inductor current is consistently positive for CCM, while for DCM the current returns to zero for each period of time. DCM causes enormous voltage stress in the circuit. Therefore, it gives an impact on electromagnetic interference (EMI) into line [11]. Voltage and current stress in DCM become excessive in high power applications, triggering the converter’s efficiency to deteriorate [12, 13]. Thus, at low power applications, usually less than 300W, DCM is commonly used, while at medium and high applications CCM becomes superior [13–15]. CCM offers lower conduction losses in the semiconductors and inductors, low output voltage ripple, lower conducted noise, lower inductor core loss, and lower inductor core loss even at low power application [13]. Despite the benefits of CCM operation, the design of the CCM controller is more arduous than DCM. Due to the nature of its topologies, DCM can attain a higher power factor as compared to CCM [16, 17]. Therefore, a simple control system is sufficient to achieve PFC.

Meanwhile, intricate control and cascade closed-loop control systems are compulsory in CCM to achieve PFC [16, 18]. In order to reduce the difficulty in controller design, there are numerous methods in reducing the order of the transfer function model reported [19–24]. The controller is optimised by the reduction order and is closely aligned with the step response and the bode plot of the original model. The dynamic response of the reduction model retains the same feature as the original model. It is important to note that most of the bridgeless SEPIC converter studies are operated in DCM with power less than 150W [6–10, 25, 26]. Based on the thorough literature review, only a few papers [27, 28] discusses bridgeless SEPIC converter studied in CCM; however, no verification on the prototype since it only discusses on simulation technique. In [29], the preliminary result of the bridgeless SEPIC converter operated in the CCM prototype is presented. However, the result produces a low power factor and low efficiency.

This paper is an extended work from [29], which describes the design and implementation of average current mode control of bridgeless SEPIC converter with CCM operated at 150 W-350 W via second-order model reduction. The second-order model reduction guarantees the simplicity of tuning the ACMC and acts efficiently as PFC. In general, the paper is organised as follows: Section 2 clarifies the operation mode of the SEPIC PFC converter in CCM. Section 3 and Section 4 discuss the proposed control method used for the converter and identify the converter parameters. In Section 5, the operation of the bridgeless SEPIC converter is validated through simulation results and hardware implementation. Section 6 summarises the main findings in this work.

## Operation Mode of SEPIC PFC Converter in CCM

**Fig 2:**
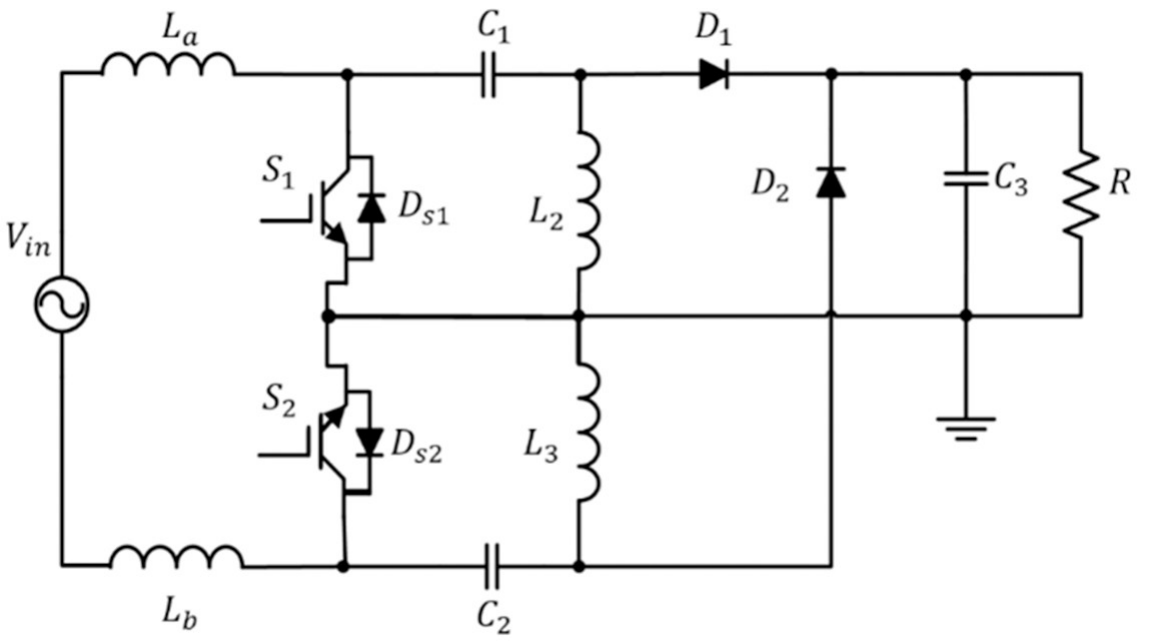
The proposed bridgeless SEPIC converter [29].

As in [10], the bridgeless SEPIC converter has been inserted with an additional inductor, *L*_*b*_ at the input side. The converter circuit, as shown in Fig 2, is proposed to work in CCM operation with an average current mode controller (ACMC). This converter is chosen due to the simplicity of the structure and fewer elements conduct at each half-cycle compared with other bridgeless SEPIC converters. The circuit consists of two IGBTs, two diodes, three capacitors, and four inductors, apart from a resistor as its load, as shown in Fig 2.

**Fig 3:**
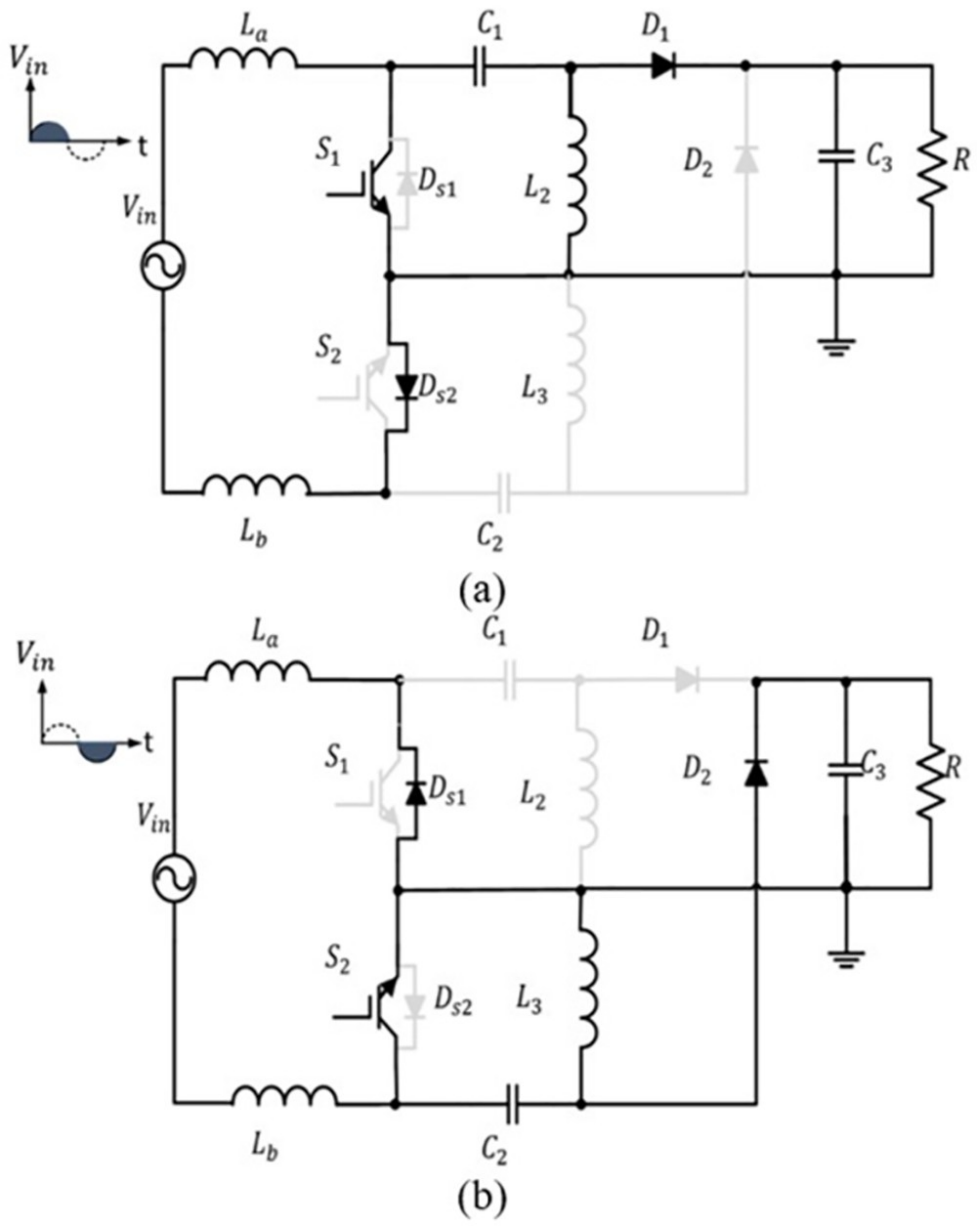
The operation of the bridgeless SEPIC converter in: (a) positive half cycle (b) negative half cycle [29].

Only nine elements conduct in the positive half cycle, which are *L*_*a*_, *L*_*b*_, *S*_1_, *D*_*s*2_, *C*_1_, *L*_2_, *D*_1_, *C*_3_*R* as shown in Fig 3(a). During the negative half cycle *L*_*a*_, *L*_*b*_, *S*_2_, *D*_*s*1_, *C*_2_, *L*_3_, *D*_2_, *C*_3_ and *R* are conducted as shown in Fig 3(b).

**Fig 4:**
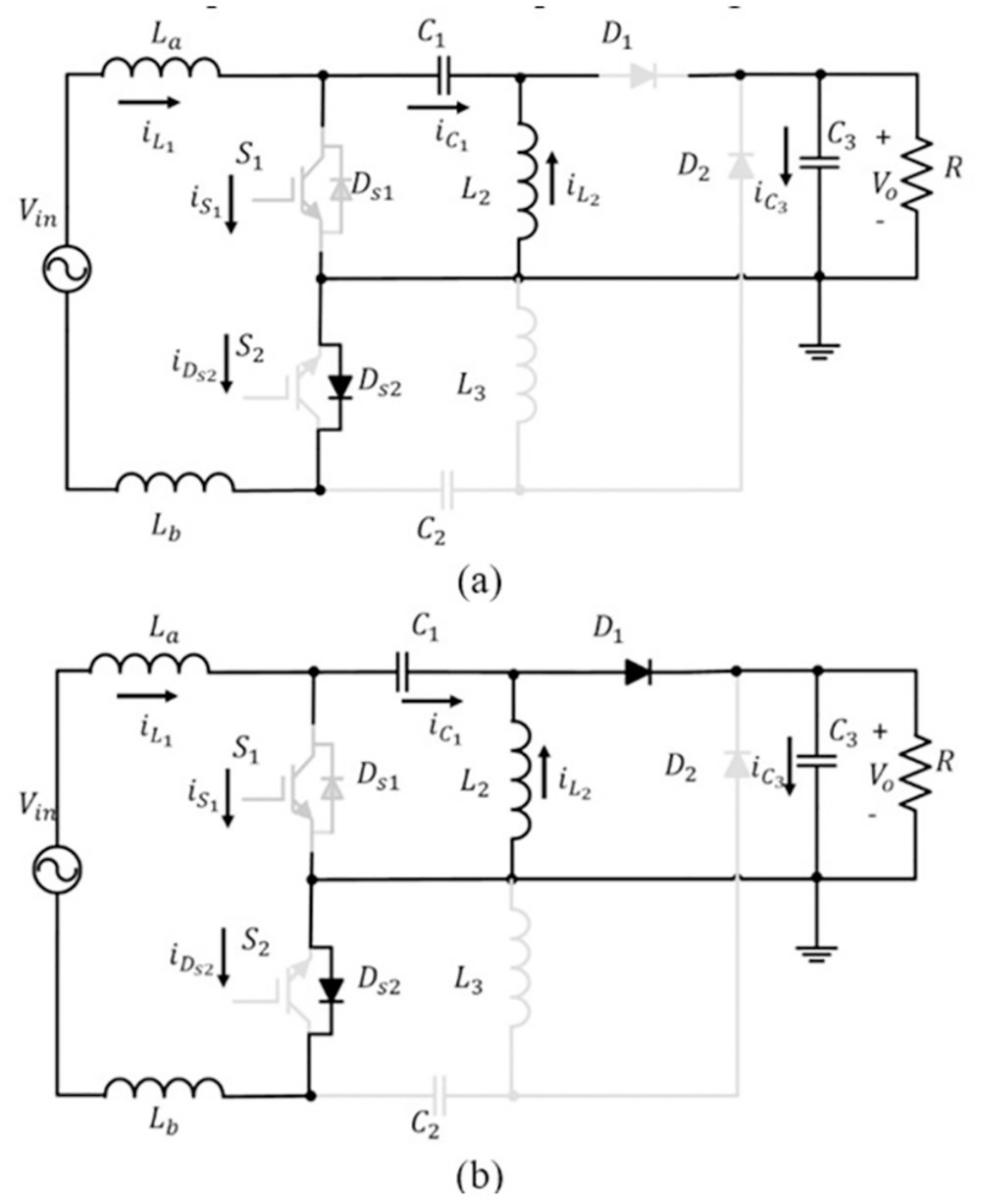
Operation of a positive half cycle in (a) mode 1 (b) mode 2 [29].

This bridgeless SEPIC converter reduces the number of conduction components through half cycle. At each half cycle of the bridgeless SEPIC converter, it acts as a basic dc-dc SEPIC converter. The previous paper [10], study on bridgeless SEPIC converter operation in DCM with a voltage controller. At each half cycle, DCM operates in three operating modes. The operation of CCM is unlike DCM, whereby for CCM, it only has two modes of operation at each half cycle. The bridgeless SEPIC converter’s operation mode is depicted in Fig 4.

**Fig 5:**
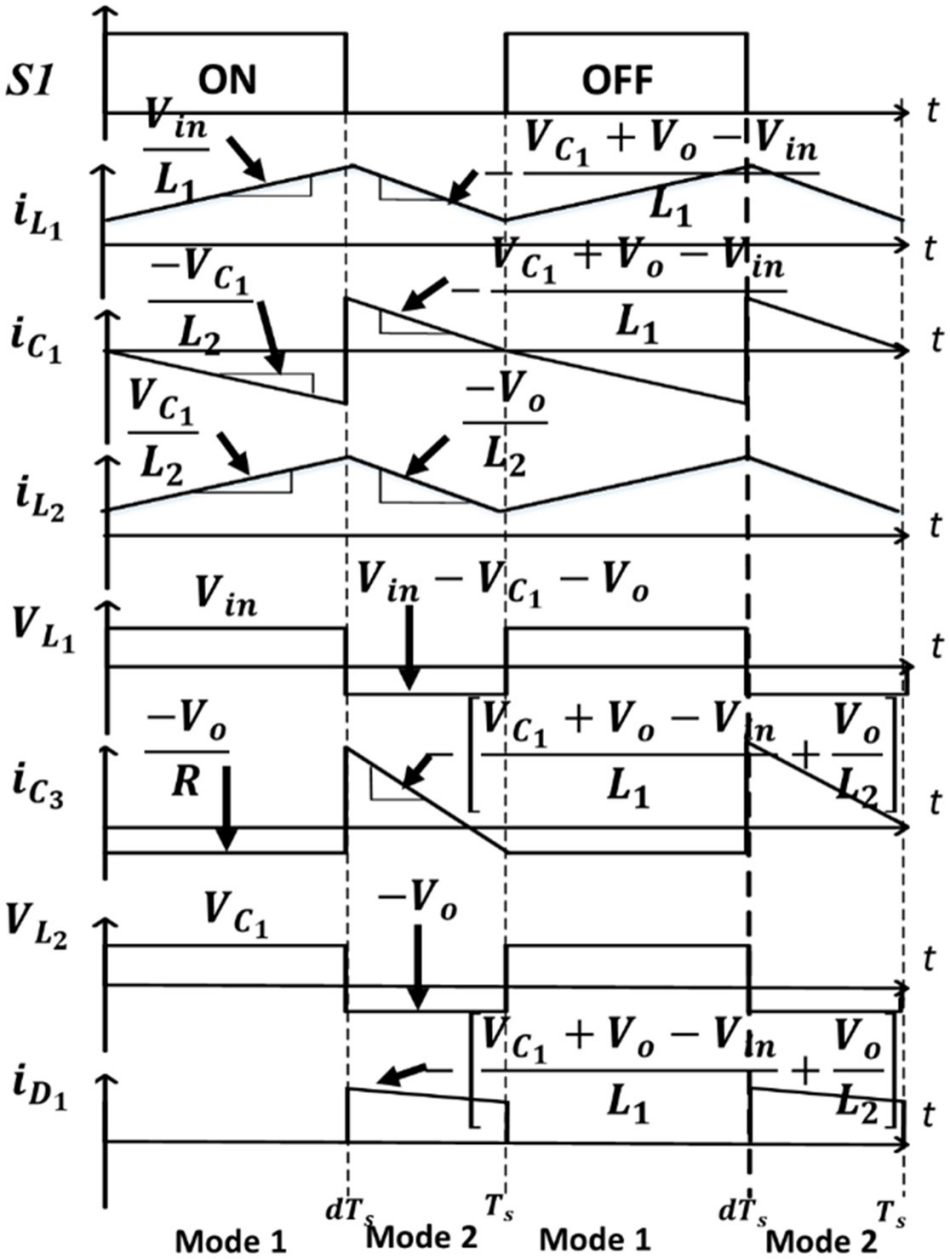
The key components waveform of the proposed converter [29].

The circuit is working in the same operation for both cycles due to the structure of the circuit, which is symmetrical in both cycles. The arrangement of element and operation is identical except for the reverse polarity of the cycle. Therefore, the converter operation is clarified only for the positive half cycle of the input voltage. The voltage and current operation waveforms of a bridgeless SEPIC converter are shown in Fig 5. To simplify circuit analysis, the sum of inductances, *L*_*a*_ and *L*_*b*_ is considered to be, *L*_1_ = (*L*_*a*_ + *L*_*b*_).

In mode 1, *S*_1_ and *D*_*s*2_ turn on by control signal and diode *D*_1_ is reversed bias. Inductor *L*_1_ charge and inductor current increase linearly concerning the relation of $\frac{V_{in}}{L_{1}}$. Capacitor *C*_1_, releases the energy to the inductor *L*_2_ and generates a current path back to *S*_1_. Since, *D*_1_ is reversed bias, the output voltage is equivalent to the output capacitor voltage. Based on the operation, the appropriate equations can be specified as follows:

The voltage across the inductor, *L*_1_

$$\begin{array}{c} L_{1}\frac{di_{L_{1}}}{dt}=V_{in} \end{array}$$ (1)

The voltage across the inductor, *L*_2_

$$\begin{array}{c} L_{2}\frac{di_{L_{2}}}{dt}=V_{C_{1}} \end{array}$$ (2)

The current across the series capacitor, *C*_1_

$$\begin{array}{c} C_{1}\frac{dv_{C_{1}}}{dt}=-i_{L_{2}} \end{array}$$ (3)

The current across the output capacitor, *C*_3_

$$\begin{array}{c} C_{3}\frac{dv_{C_{3}}}{dt}=-\frac{V_{o}}{R} \end{array}$$ (4)

In mode 2, *S*_1_ turns off and *D*_1_ forwards bias, which allows current flow through it. Due to the discharging process through the *C*_1_, *C*_3_ and load, current across *L*_2_ decrease linearly. Based on the operation, the important equations can be specified as follow:

The voltage across the inductor, *L*_1_

$$\begin{array}{c} L_{1}\frac{di_{L_{1}}}{dt}=V_{in}-V_{C_{1}}-V_{o} \end{array}$$ (5)

The voltage across the inductor, *L*_2_

$$\begin{array}{c} L_{2}\frac{di_{L_{2}}}{dt}=-V_{C_{3}} \end{array}$$ (6)

The current across the series capacitor, *C*_1_

$$\begin{array}{c} C_{1}\frac{dv_{C_{1}}}{dt}=i_{L_{1}} \end{array}$$ (7)

The current across the output capacitor, *C*_3_

$$\begin{array}{c} C_{3}\frac{dv_{C_{3}}}{dt}=i_{L_{1}}+i_{L_{2}}-\frac{V_{o}}{R} \end{array}$$ (8)

## Proposed control method

In order to implement the bridgeless SEPIC converter working in CCM, the basic proportional-integral (PI) controller is used as a compensator in the ACMC. In designing the controller, the transfer function based on a mathematical model is necessary in order to simplify control tuning. The state-space averaging modelling technique [30–32] is employed in the bridgeless SEPIC converter in CCM operation. Applying the KCL and KVL of the bridgeless converter circuit during the turn ON and OFF state of *S*_1_ as per Eqs (1)–(8). The average model of the bridgeless SEPIC converter is:

$$\begin{array}{c} \begin{array}{c} L_{1}\frac{di_{L_{1}}}{dt}=V_{in}-\left(1-d\right)\left(V_{c_{1}}+V_{o}\right)\\ L_{2}\frac{di_{L_{2}}}{dt}=dV_{C_{1}}-\left(1-d\right)V_{o}\\ C_{1}\frac{dv_{C_{1}}}{dt}=\left(1-d\right)i_{L_{1}}-di_{L_{2}}\\ C_{3}\frac{dv_{0}}{dt}=\left(1-d\right)\left(i_{L_{1}}-i_{L_{2}}\right)-\frac{V_{0}}{R} \end{array} \end{array}$$ (9)

Steady-state dc model:

$$\begin{array}{c} \;\begin{array}{c} 0=AX+BU\\ Y=CX \end{array} \end{array}$$ (10)

AC small-signal model:

$$\begin{array}{c} \;\begin{array}{c} \tilde{x}=A\tilde{x}+B\tilde{u}+B_{d}\tilde{d}\\ \tilde{y}=C\tilde{x} \end{array} \end{array}$$ (11)

$\tilde{y}$ -vector of output system $\left(i_{L_{1}},v_{o}\right)$

A- State vector $\left(\tilde{i_{L_{1}}},\tilde{i_{L_{2}}},\tilde{v_{c_{1}}},\tilde{v_{o}}\right)$

B-Input matrix

C- Matrix, which connects the output to the state variable

$\tilde{u}$ - Input variable (*V*_*in*_)

$B_{d}.\tilde{d}$ - Duty ratio variation for CCM [31]

Based on Eqs 9, 10, and 11, the average matrices for the steady state and linear small signal state space equations of bridgeless SEPIC converters are as follows: -

$$\begin{array}{c} \frac{d}{dt}\lceil \begin{array}{c} \tilde{i_{L_{1}}}\\ \;\tilde{i_{L_{2}}}\\ \tilde{v_{C_{1}}}\\ \tilde{V_{0}} \end{array}\rceil =A.\lceil \begin{array}{c} \tilde{i_{L_{1}}}\\ \;\tilde{i_{L_{2}}}\\ \tilde{v_{C_{1}}}\\ \tilde{V_{0}} \end{array}\rceil +B_{d}.V_{in}+B.\tilde{d} \end{array}$$ (12)

$$\begin{array}{c} \frac{d}{dt}\lceil \begin{array}{c} \tilde{i_{L_{1}}}\\ \tilde{V_{0}} \end{array}\rceil =C.\lceil \begin{array}{c} \tilde{i_{L_{1}}}\\ \tilde{i_{L_{2}}}\\ \tilde{v_{C_{1}}}\\ \tilde{V_{0}} \end{array}\rceil \end{array}$$ (13)

where:

$$A=\lceil \begin{array}{cccc} 0 & 0 & -\frac{\left(1-d\right)}{L_{1}} & -\frac{\left(1-d\right)}{L_{1}}\\ 0 & 0 & \frac{d}{L_{2}} & -\frac{\left(1-d\right)}{L_{2}}\\ \frac{\left(1-d\right)}{C_{1}} & \frac{d}{C_{1}} & 0 & 0\\ \frac{\left(1-d\right)}{C_{3}} & \frac{\left(1-d\right)}{C_{3}} & 0 & -\frac{1}{RC_{3}} \end{array}\rceil ,B=\lceil \begin{array}{c} \frac{V_{O}}{L_{1}d}\\ \frac{V_{O}}{L_{2}d}\\ -\frac{V_{O}}{RC_{3}\left(1-d\right)}\\ -\frac{V_{O}}{RC_{3}\left(1-d\right)} \end{array}\rceil ,B_{d}=\lceil \begin{array}{c} \frac{1}{L_{1}}\\ 0\\ 0\\ 0 \end{array}\rceil ,\text{C}=\lceil \begin{array}{cccc} 1 & 0 & 0 & 0\\ 0 & 0 & 0 & 1 \end{array}\rceil ,$$

Eqs (12) and (13) are derived based on ac perturbation. The bridgeless SEPIC converter’s transfer function is extracted using the Laplace transformation in (12) and (13).

$$\begin{array}{c} Y\left(s\right)=\lceil \begin{array}{c} i_{L_{1}}\left(s\right)\\ v_{o}\left(s\right) \end{array}\rceil =C{\left(sI_{4}-A\right)}^{-1}B_{d}.d\left(s\right)+C{\left(sI_{4}-A\right)}^{-1}.BV_{in} \end{array}$$ (14)

where *I*_4_ is a unity matrix, the inner loop and outer loop transfer functions are as follows, based on the expansion and solution of Eq (9): -

$$\begin{array}{c} \;\begin{array}{c} G_{vi}=\frac{i_{L_{1}}\left(s\right)}{d(s)}\\ =\frac{a_{x}\left(s^{3}+a_{1}s^{2}+a_{2}s+a_{3}\right)}{s^{4}+b_{1}s^{3}+b_{2}s^{2}+b_{3}s+b_{4}}\; \end{array} \end{array}$$ (15)

$$\begin{array}{c} \;\begin{array}{c} G_{id}=\frac{v_{o}\left(s\right)}{i_{L_{1}}\left(s\right)}\\ =\frac{-a_{y}\left(s^{3}-a_{4}s^{2}+a_{5}s-a_{6}\right)}{s^{3}+a_{1}s^{2}+a_{2}s+a_{3}}\; \end{array} \end{array}$$ (16)

with:

$$\begin{array}{cc} a_{1}= & \frac{1}{RC_{2}\left[d\left(\frac{1+C_{3}}{C_{1}}\right)+1\right]},a_{2}=\frac{d}{L_{2}C_{1}}\left[1+\left(\frac{L_{2}}{C_{3}R^{2}}\right)\right],a_{3}=2d\left[RL_{1}C_{3}C_{1}\right],\\ a_{4}= & {\left(1-d\right)}^{2}\frac{R}{L_{1}d}\left[1+\frac{L_{1}}{L_{2}}\right],a_{5}=\frac{d}{L_{2}C_{1}},a_{6}=\frac{R{\left(1-d\right)}^{2}}{dL_{1}L_{2}C_{1}},b_{1}=\frac{1}{RC_{2}},\\ b_{2}= & \frac{1}{L_{1}C_{1}}[\left(\frac{L_{1}}{L_{2}}d^{2}\left(\frac{C_{1}}{C_{3}}{\left(1-d\right)}^{2}\right)+\left({\left(1-d\right)}^{2}\frac{C_{1}}{C_{3}}\right)\right],\\ b_{3}= & \frac{1}{\left(RL_{1}C_{1}C_{3})\right)}\left[\left(\frac{L1}{L_{2}}d^{2}\right)+{\left(1-d\right)}^{2}\right],b_{4}=\frac{{\left(1-d\right)}^{2}}{L_{1}L_{2}C_{1}C_{3}},a_{x}=\frac{V_{O}}{L_{1}d},\\ a_{y}= & -\frac{L_{1}d}{RC_{3}\left(1-d\right)} \end{array}$$

The design of the controller for a bridgeless SEPIC converter is quite a challenging task since the transfer function is in 4^*th*^ order. By using model order reduction via improved Padé approximations and Routh Hurwitz array in [19–22], the 4^*th*^ order transfer function is now reduced to 2^*nd*^ order to simplify the tuning of the controller:

The 2^*nd*^ order reduction transfer function is in the form of: -

$$\begin{array}{c} R_{2}=\frac{e_{1}+e_{2}s}{f_{1}+f_{2}s+f_{3}s^{2}} \end{array}$$ (17)

**Fig 6:**
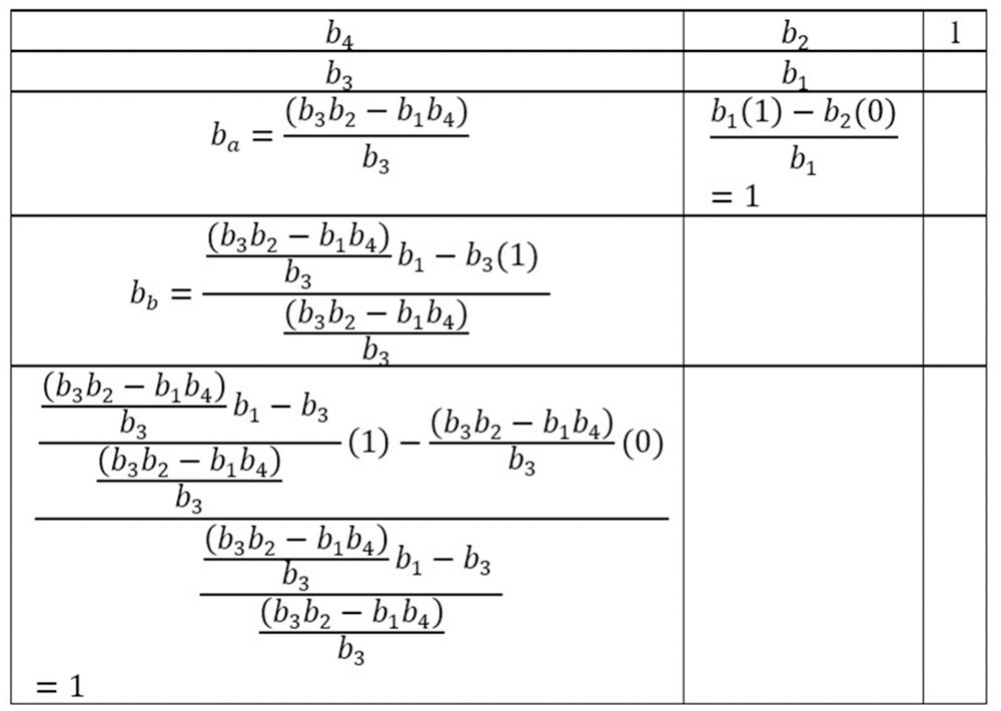
The converse Routh array for the denominator of the original inner loop transfer function, *G*_*id*_.

The original transfer function’s denominator is reduced to 2^*nd*^ order by using the Routh Hurwitz array. The denominator of the original inner loop transfer function, *G*_*id*_, is arranged using Eq 15 in the converse Routh array as in Fig 6.

By taking the first three values on the first column of the Routh array, the denominator of the 2^*nd*^ order transfer function is

$$\begin{array}{c} D_{id}=b_{a}s^{2}+b_{3}s+b_{4} \end{array}$$ (18)

By normalising *D*_*id*_, produces

$$\begin{array}{c} D_{id}=s^{2}+\frac{b_{3}}{b_{a}}s+\frac{b_{4}}{b_{a}} \end{array}$$ (19)

The reduced order of numerator coefficient is attained via improved Padé approximation of the original transfer function, *G*_*id*_

$$\begin{array}{c} \frac{a_{x}\left(s^{3}+a_{1}s^{2}+a_{2}s+a_{3}\right)}{\left(s^{4}+b_{1}s^{3}+b_{2}s^{2}+b_{3}s+b_{4}\right)}=g_{0}+g_{1}s+g_{2}s^{2} \end{array}$$ (20)

$$\begin{array}{c} g_{0}=\frac{a_{x}a_{3}}{b_{4}} \end{array}$$ (21)

$$\begin{array}{c} g_{1}=\frac{1}{b_{4}}\left(a_{x}a_{2}-b_{3}g_{0}\right) \end{array}$$ (22)

$$\begin{array}{c} g_{2}=\frac{1}{b_{4}}\left(a_{x}a_{1}-b_{2}g_{0}-b_{3}g_{1}\right) \end{array}$$ (23)

$$\begin{array}{c} e_{1}=f_{1}g_{0} \end{array}$$ (24)

$$\begin{array}{c} e_{2}=f_{1}g_{1}+f_{2}g_{0} \end{array}$$ (25)

By substituting Eqs (21–23) into Eqs (24–25) and solving to the 2^*nd*^ order reduction form in Eq (17), the reduce order transfer function of the inner loop, *R*_*id*_ are:

$$\begin{array}{c} R_{id}=\frac{h_{1}+h_{2}s}{n_{1}+n_{2}s+n_{3}s^{2}} \end{array}$$ (26)

where,

$$h_{1}=\frac{a_{x}a_{3}}{b_{a}},h_{2}=\frac{1}{b_{a}}\left(a_{x}a_{1}-b_{2}g_{0}-b_{3}g_{1}+\frac{b_{3}a_{x}a_{3}}{b_{4}}\right),n_{1}=1,n_{2}=\frac{b_{3}}{b_{a}},n_{3}=\frac{b_{4}}{b_{a}}$$

Solving the similar step to the outer loop *G*_*vi*_ transfer function, yield

$$\begin{array}{c} R_{vi}=\frac{p_{1}+p_{2}s}{q_{1}+q_{2}s+q_{3}s^{2}} \end{array}$$ (27)

where,

$$p_{1}=\frac{a_{y}a_{6}}{a_{2}},p_{2}=\frac{1-a_{y}a_{3}a_{5}-a_{y}a_{2}a_{6}}{a_{z}}+\frac{a_{y}a_{z}a_{6}}{b_{4}},q_{1}=\frac{a_{3}}{a_{z}},q_{2}=\frac{a}{a_{z}},q_{3}=1,a_{z}=\frac{a_{2}a_{1}-a_{3}}{a_{2}}$$

## Converter parameters

The proposed circuit is based on the requirements specifications listed in Table 1. The bridgeless SEPIC converter is designed to operate in CCM mode with all parameters.

**Table 1:** The propose converter parameter.

Parameter	Value
Line Frequency, *f*_*L*_	50 Hz
Switching Frequency, *f*_*s*_	20 KHz
Input Voltage, *V*_*in*_	110—220 *V*_*rms*_
Output Voltage, *V*_*o*_	100 *V*_*dc*_
Power Output, *P*_*o*_	100—350 W

### The boundary between CCM and DCM

All parameters are designed to meet the anticipated performance of the converter based on the following condition: Based on [33, 34], the voltage conversion ratio,

$$\begin{array}{c} M=\frac{V_{o}}{V_{in}} \end{array}$$ (28)

The boundary between CCM and DCM can be determined using the following formula:

Critical conduction parameter,

$$\begin{array}{c} K_{crit}=\frac{1}{2{\left(M+1\right)}^{2}} \end{array}$$ (29)

It is compulsory to choose the conduction parameter, *K* > *K*_*crit*_ to guarantee the circuit operates in CCM operation otherwise, it will operate in DCM. In this converter, the *K* = 0.8 is selected.

### Design of inductors

The bridgeless SEPIC converter equivalent inductances are determined as follows:

The equivalent inductor in series,

$$\begin{array}{c} L_{eq}=\frac{KR}{2f_{s}} \end{array}$$ (30)

$$\begin{array}{c} L_{eq}=\frac{L_{1}L_{3}}{L_{1}+L_{3}} \end{array}$$ (31)

$$\begin{array}{c} L_{eq}=\frac{L_{1}L_{3}}{L_{1}+L_{3}} \end{array}$$ (32)

The inductor *L*_1_ values are calculated using the inductor current ripple equation as below

$$\begin{array}{c} L_{1}=\frac{V_{in}D}{\Delta i_{L_{1}}f_{s}} \end{array}$$ (33)

The maximum inductor current ripple is calculated using the permitted ripple of 20% of input current. Value of *L*_3_ can be computed based on Eqs (18–20) where R is the load resistance, $\Delta i_{L_{1}}$ is the percentage of input current ripple and *d* is duty ratio (choose the worst case). The value of *L*_*a*_ = *L*_*b*_ and *L*_2_ = *L*_3_ because the structures of the converter are symmetrical.

### Design of the capacitors

The intermediate capacitor, *C*_1_ is designed to adapt the input voltage pattern during the switching cycle and provide constant voltage during the line cycle. In order to avoid input current oscillations at each half cycle, the resonant frequency between (*L*_1_, *C*_1_, *L*_3_, and *C*_3_) must be set higher than the line frequency but lower than the switching frequency in order to sustain constant voltage throughout the switching time.

$$\begin{array}{c} C_{1}=\frac{1}{{f_{r}}^{2}\left(L_{1}L_{3}\right)} \end{array}$$ (34)

where *f*_*r*_ is the resonant frequency.

The output capacitor, *C*_3_ is used to minimise the output voltage ripple of the converter. It is necessary to be designed large enough to decrease output voltage ripple based on 20% permitted percentage ripple.

$$\begin{array}{c} C_{3}=\frac{D}{R\Delta V_{o}f_{s}} \end{array}$$ (35)

where Δ*V*_*o*_ is the voltage output ripple percentage. Table 2 tabulates the proposed converter parameter for bridgeless SEPIC converter based on the calculation value.

**Table 2:** The propose converter parameter.

Parameter	Value
Input inductor *L*_*a*_,*L*_*b*_	5 mH
Intermediate inductor, *L*_2_,*L*_3_	0.178 mH
Intermediate Capacitor, *C*_1_,*C*_2_	2*μF*
	EPCOS MKP B32774D0205K000
Output Capacitor, *C*_3_	4800*μF*
	104 PHL-ST
Output Resistor, *R*	29–67Ω
*S*_1_,*S*_2_	2MBI100U4A-120
*D*_1_,*D*_2_	IDH20G120C5

## Design of average current mode controller

**Fig 7:**
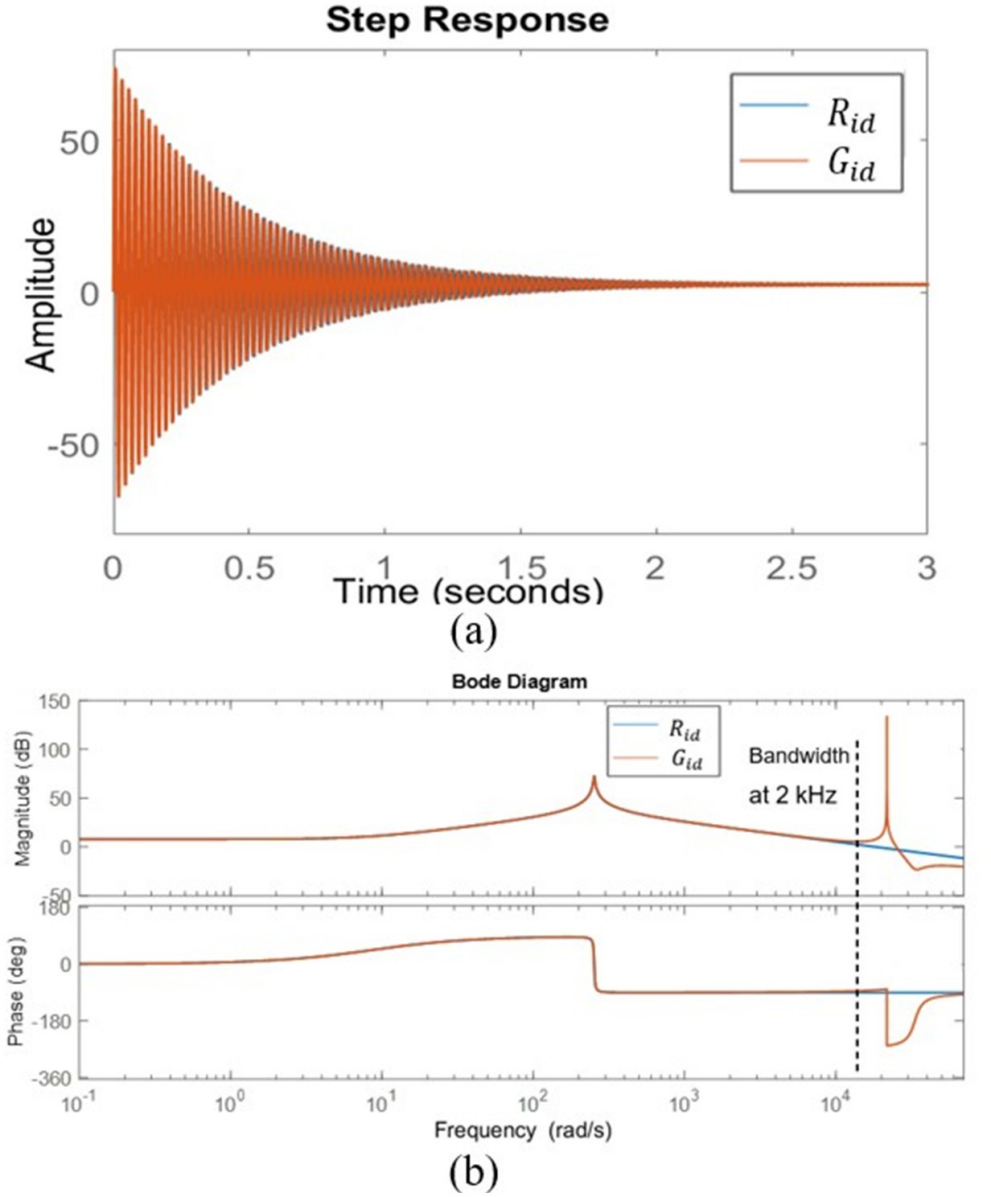
The original model of the inner loop, *G*_*id*_ and second order model of the inner loop, *R*_*id*_ (a) Step response (b) Bode plot.

**Fig 8:**
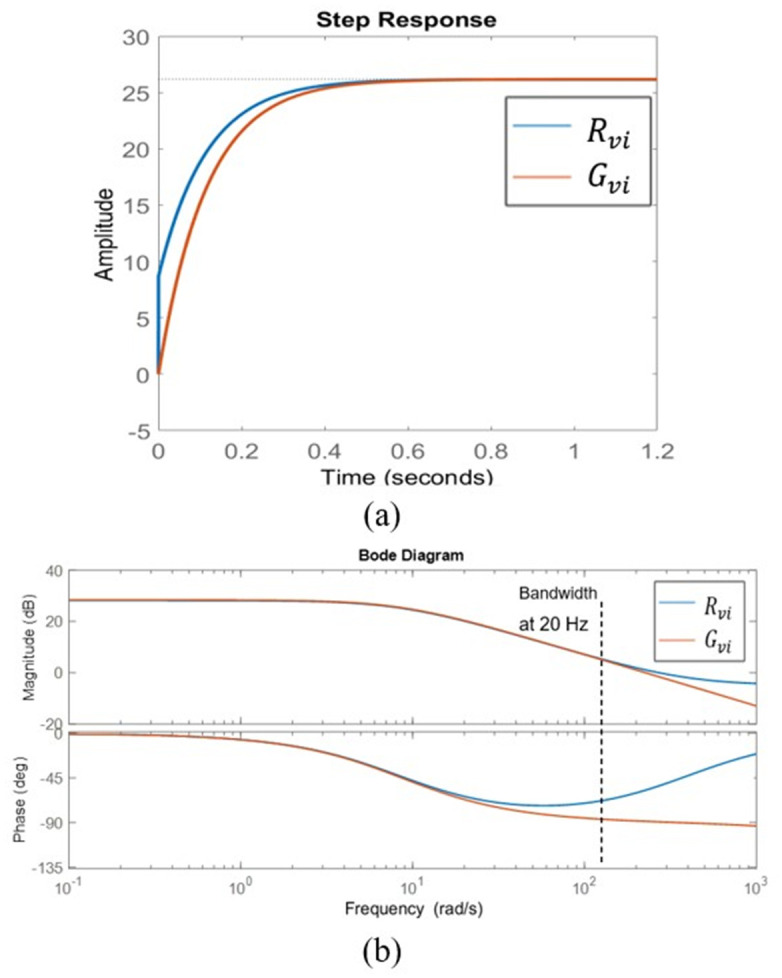
The original model of the outer loop, *G*_*vi*_ and second order model of the outer loop, *R*_*vi*_ (a) Step response (b) Bode plot.

By substituting the parameter value in Tables 1 and 2 of the bridgeless SEPIC converter in section 4 into Eqs (26) and (27). The step response and bode plot of the bridgeless SEPIC converter for the original model and 2^*nd*^ order reduction for the inner loop and outer loop as are shown in Figs 7 and 8. The step response of the inner loop reduction model, *R*_*id*_ is identical to the unstable step response found in the original model, *G*_*id*_ as shown in Fig 7(a) while for the outer loop reduction model, *R*_*vi*_ the step response is in good agreement with the original model as witnessed in Fig 8(a).

It can be seen that *R*_*id*_ tracks the unstable condition and inherit all the characteristics of *G*_*id*_. The inner loop reduction model, *R*_*vi*_ response resembles the original model, *G*_*vi*_ in both steady and transient states. *R*_*id*_ and *R*_*vi*_ transfer function is less complicated, and the complexity during the transfer function tuning process is massively diminished. The bode plot demonstrates that due to the characteristics of Padé approximations, the reduced order, *R*_*id*_ and *R*_*vi*_ are accurate up to 2 kHz and 20 Hz frequency, respectively. These boundary frequencies are important when designing the controller. At high frequencies, the difference in error between the original and reduction model systems is marginal. It is important to note that the Padé approximations provide an excellent job of capturing low frequency dynamics while the accuracy at high frequency increases as the reduction order increases [23]. The step response of both transfer functions inherits the same trait, dynamic response, and exhibits the same behaviors as the original model. The approximation of the converter system in second order is acceptable and most of the system property is preserved. It is simple and effective in simplifying the high-order transfer function controller design.

**Fig 9:**
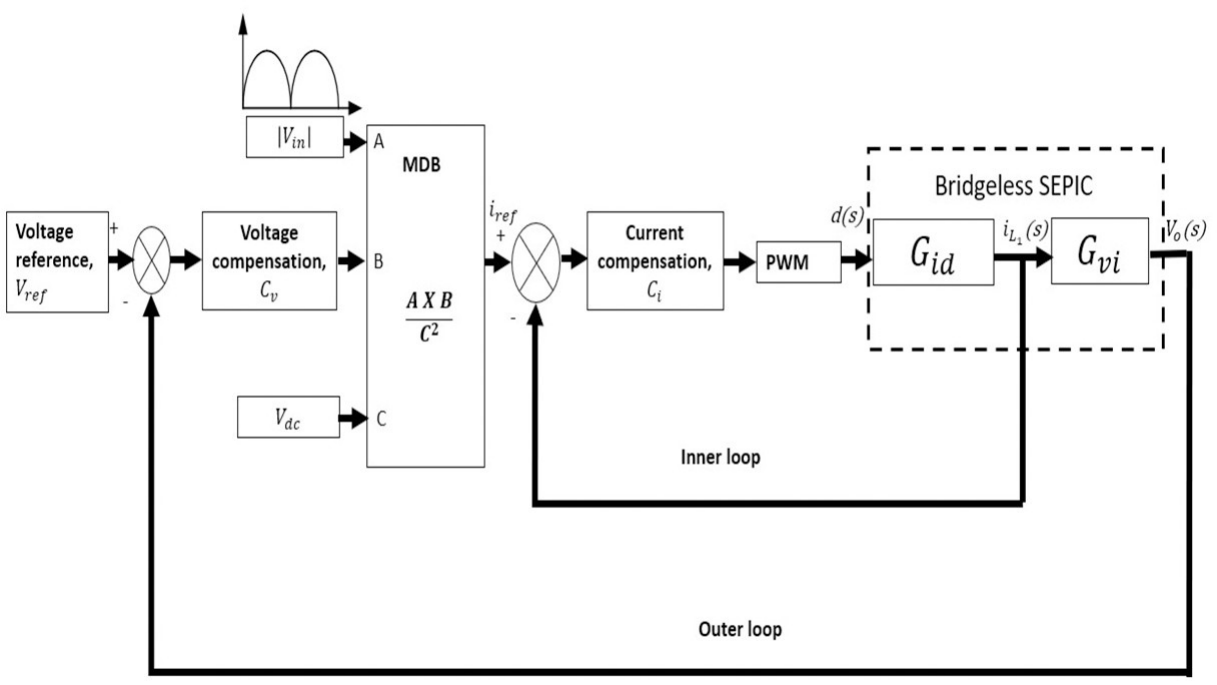
The proposed ACMC for bridgeless SEPIC using the PI controller.

The proposed controller for the bridgeless SEPIC converter using ACMC is illustrated in Fig 9. ACMC function to force the input current to track the reference current. To reshape the input current, two cascaded loops to perform as a suitable controller are required. The inner loop is designed to control the current and the outer loop to control the voltage. To simplify the proposed controller, Eqs (26) and (27) are used as inner loop and outer loop transfer functions to design the ACMC of the bridgeless SEPIC converter.

The outer loop with a slower response act to provide an error between the reference voltage and the actual output voltage to generate the input current reference for the inner loop. This primary loop function is to adjust and sustain voltage to the desired set point.

The inner loop performs as current shaping for the input current. The actual input current is commanded to track sinusoidal reference current, *i*_*ref*_ that leads to enhanced input power factor. The current loop compensator adjusts the duty ratio in response to changes in input current, ensuring that the output voltage remains constant. The inner loop output is compared to the sawtooth carrier to produce a pulse width modulation (PWM) signal with a fixed frequency.

The most critical component in attaining the ACMC is the multiplier or multiplication and dividing signal (MDB). MDB role is to accomplish the input current reference signal to feed in the loop. A high power factor can be achieved as the input voltage waveform, and the reference current waveform is identical depending on the multiplier technique [35, 36]. MDB is mainly composed of a PI voltage error, a multiplier, and a division of a rectified voltage input variable |*V*_*i*_*n*| with an average component of input rectified voltage, *V*_*dc*_. This controller requires both voltage and current sensors to feed MDB.

**Fig 10:**
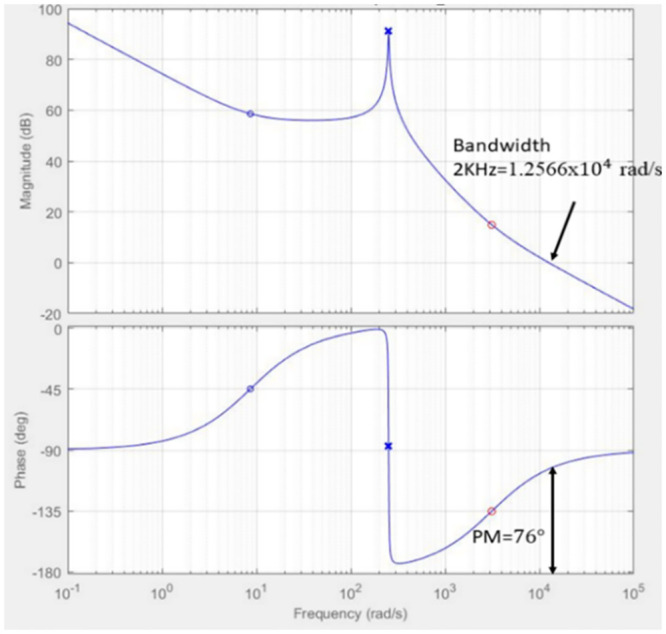
Bode plot of the inner loop with PI compensator.

**Fig 11:**
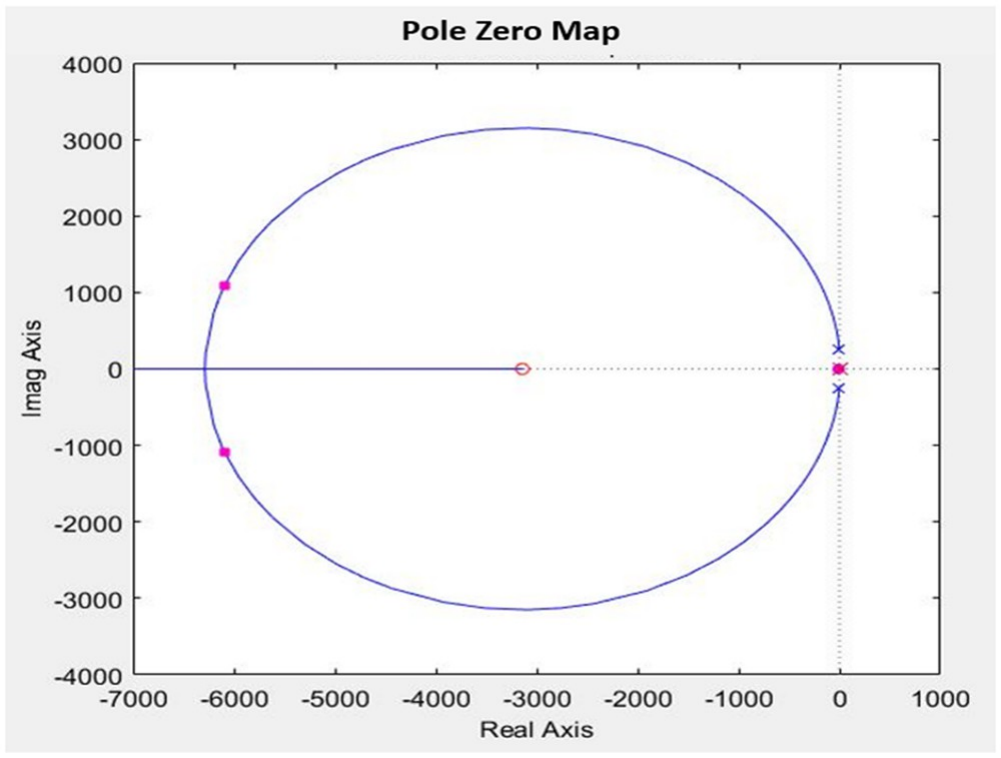
Pole Zero plot of the inner loop with PI compensator.

The ACMC controller is designed to achieve the preferred PI controller for both the inner and outer loops through the SISO tool in MATLAB/Simulink. The inner loop bandwidth is designed to be a decade lower (2 kHz) than the switching frequency (20 kHz) using the transfer function according to Eq (14) to guarantee the high reliability of the control signal. Hence, the gain for the PI controller for the inner loop is set to *K*_*p*_ = 0.66715 and *K*_*i*_= 3138, resulting in a phase margin of 76 degrees with bandwidth at 1.2566*x*10^4^*rad*/*s* as can be seen in Fig 10. This value is chosen due to the stability and performance of the controller during the transient response, as well as the ability to achieve the desired bandwidth for the control converter. Both zeros and poles for the transfer function are in the left-hand plane (LHP) which shows the stability of the controller as in Fig 11.

**Fig 12:**
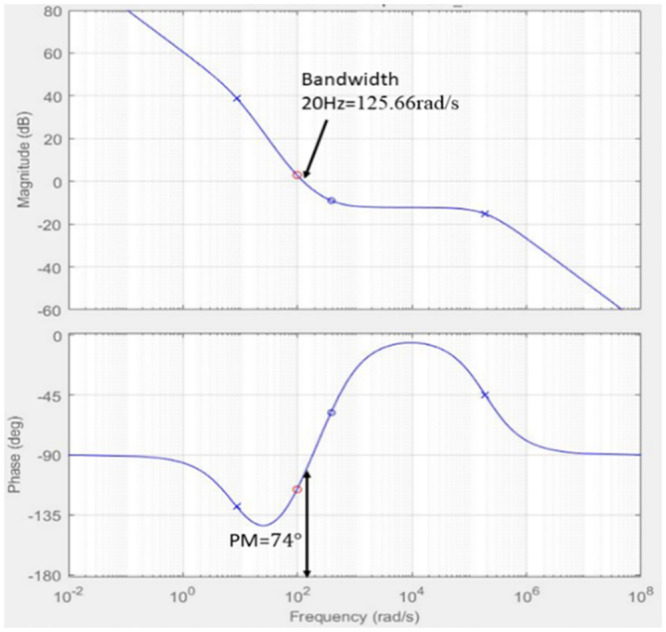
Bode plot of the outer loop with PI compensatorr.

**Fig 13:**
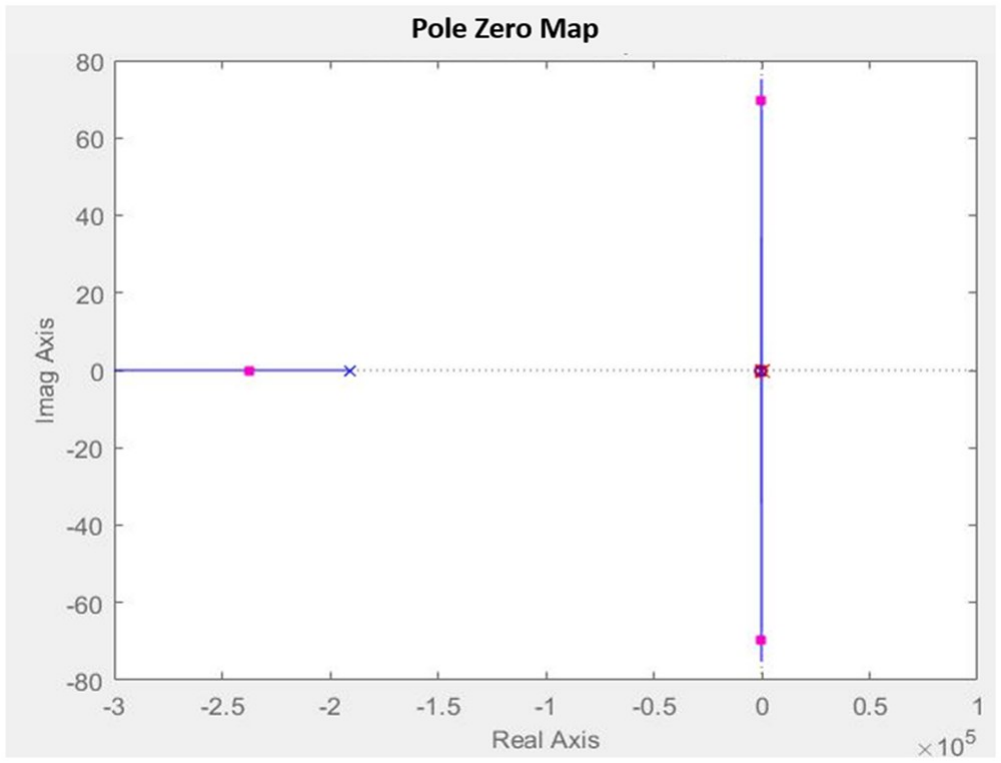
Bode plot of the outer loop with PI compensator.

The outer loop bandwidth is assigned to 20 Hz below the line frequency to prevent oscillation in the input current based on the transfer function in the Eq (26). For the outer loop, the gain for the PI controller is set to *K*_*p*_ = 0.37001 and *K*_*i*_= 17.7822, resulting in a phase margin of 74 degrees with bandwidth at 125.66 rad/s, as shown in Fig 12. Both zeros and poles for the transfer function are in the left-hand plane (LHP) which shows the stability of the controller as in Fig 13. The controller is designed to produce a quick dynamic response and low output voltage ripple.

## Simulation and experimental results

**Fig 14:**
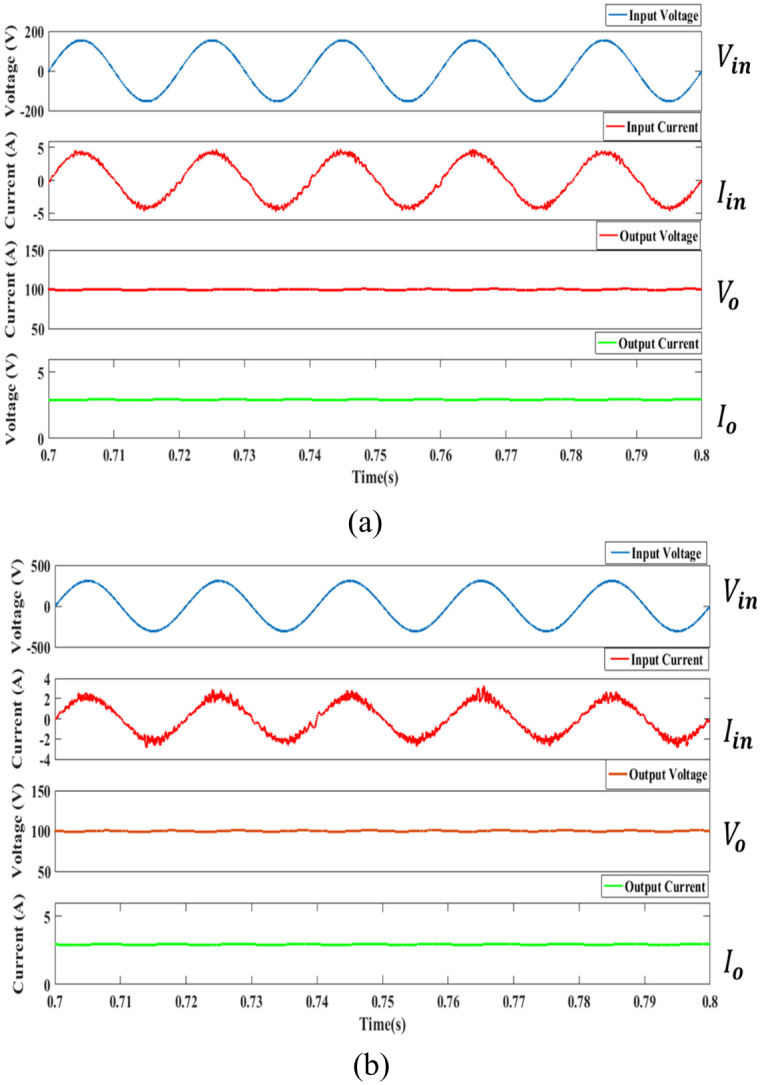
The simulated waveform for the converter at rated load (a) 110 *V*_*in*_*rms* (b) 220 *V*_*in*_*rms*.

The simulation to evaluate the proposed controller was carried out using Matlab/Simulink and the parameters described in Table 2. Fig 14 shows the simulation result at a steady state for 110 *V*_*in*_*rms* and 220 *V*_*in*_*rms* at the rated load.

It can be seen that the input voltage and the input current are in phase. The output voltage has accomplished the anticipated 100 V while the output current achieves almost 3.5 A for both input voltage. The output voltage and output current ripple for high voltage input generate more ripple than low voltage input based on the simulation outcome. The sinusoidal waveform of the input current for low input voltage shape is less distorted compared to the low voltage input. The average output power successfully achieves 350 W as anticipated. Based on the simulation result, the proposed controller shows a satisfactory result in PFC at CCM operation.

**Fig 15:**
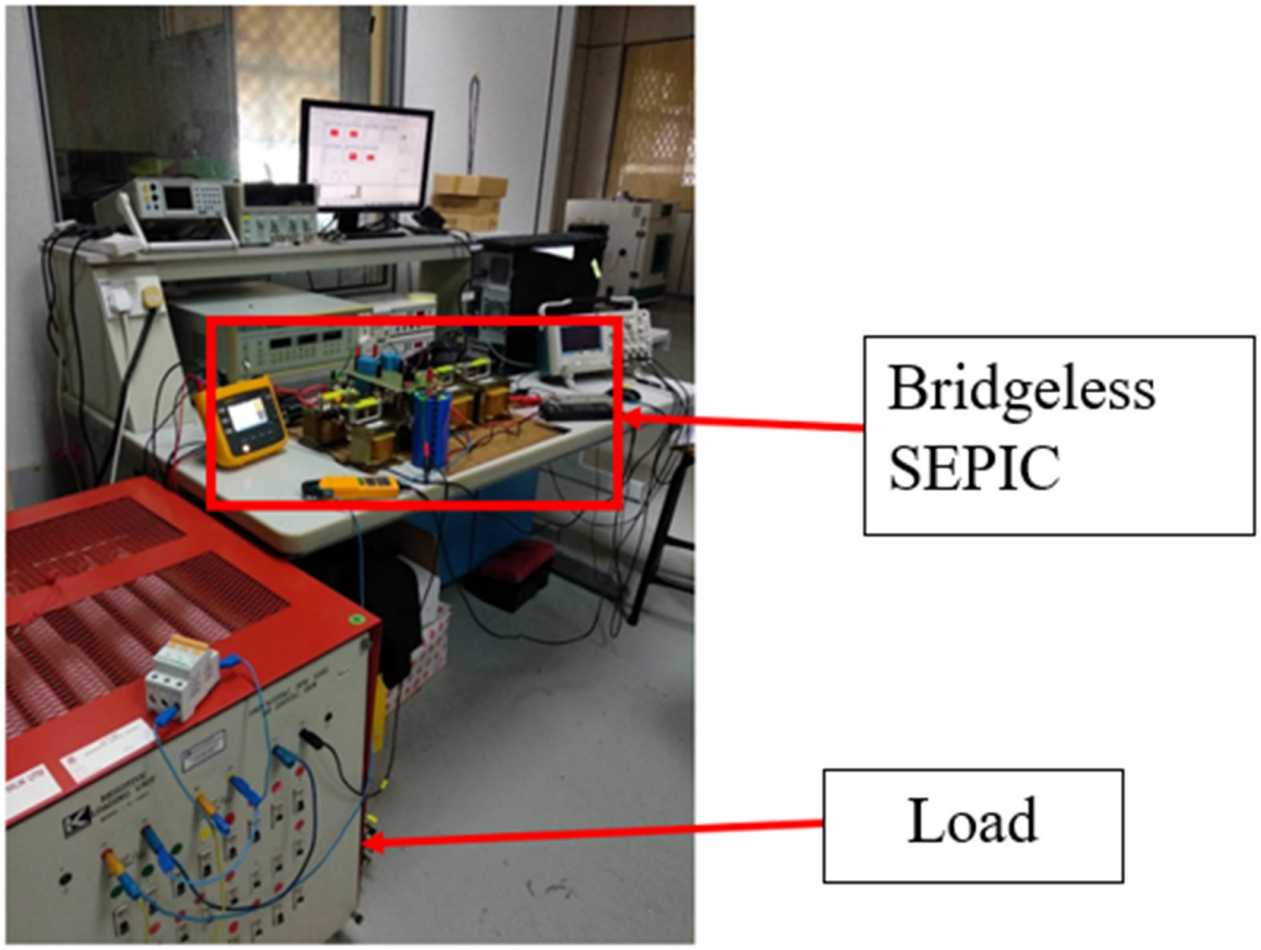
The prototype of bridgeless SEPIC.

A bridgeless SEPIC converter prototype as shown in Fig 15, is developed to validate the proposed controller and simulation results in CCM. The construction of the prototype setup as per the specifications as in the simulation. The ac input filter has been added in order to maintain a good ac supply to the prototype. The controller is implemented using a basys 3 field-programmable gate array (FPGA) and DS1104 digital signal processing and control engineering (dSPACE) controller board to achieve exact sampling time. The dSPACE control desk is used as an ACMC controller interface to detect input current, input voltage and output voltage as indicated. All the sensing input provides feedback to the dSPACE control desk, and all calculation of MDB calculation is performed to generate a reference current to the converter. However, dSPACE limited the lower sampling time by deferring the prototype performance and consistency from simulation. A low sampling time of 20 *μs* is achieved by combining dSPACE and basys3 FPGA.

**Fig 16:**
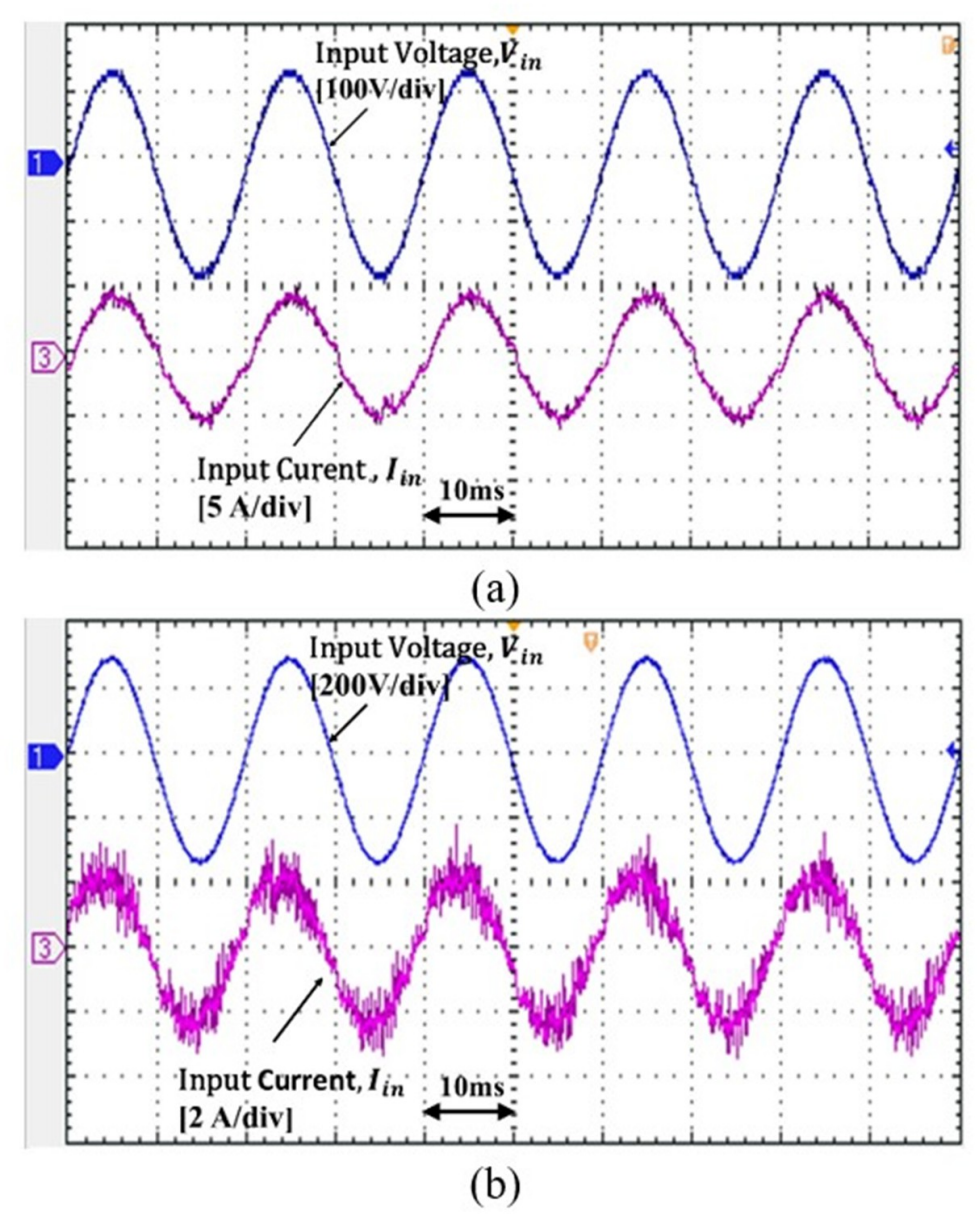
Input voltage *V*_*in*_ and input current *I*_*in*_ for 350W at (a) P = 110 *V*_*in*_*rms*, (b) P = 220 *V*_*in*_*rms*.

Fig 16 shows the experiment result of the input line voltage and input line current for 110 *V*_*in*_*rms* and 220 *V*_*in*_*rms* at 350 W. The controller accomplishes to shape the sinusoidal input current as predicted to achieve PFC at CCM operation.

**Fig 17:**
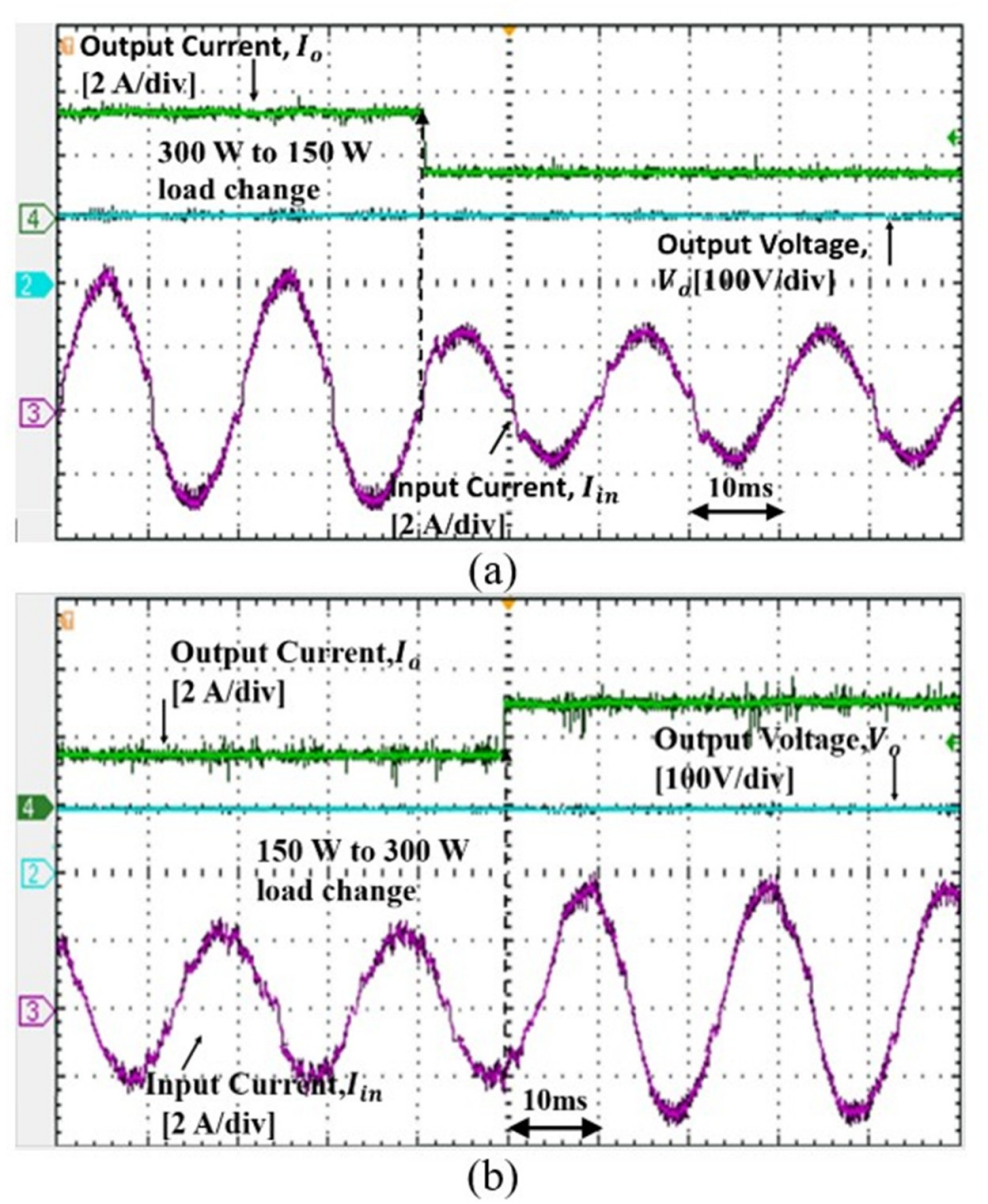
Converter response to the load change for 110 *V*_*in*_*rms* (a) from 300 W to 150 W (b) from 150 W to 300 W.

**Fig 18:**
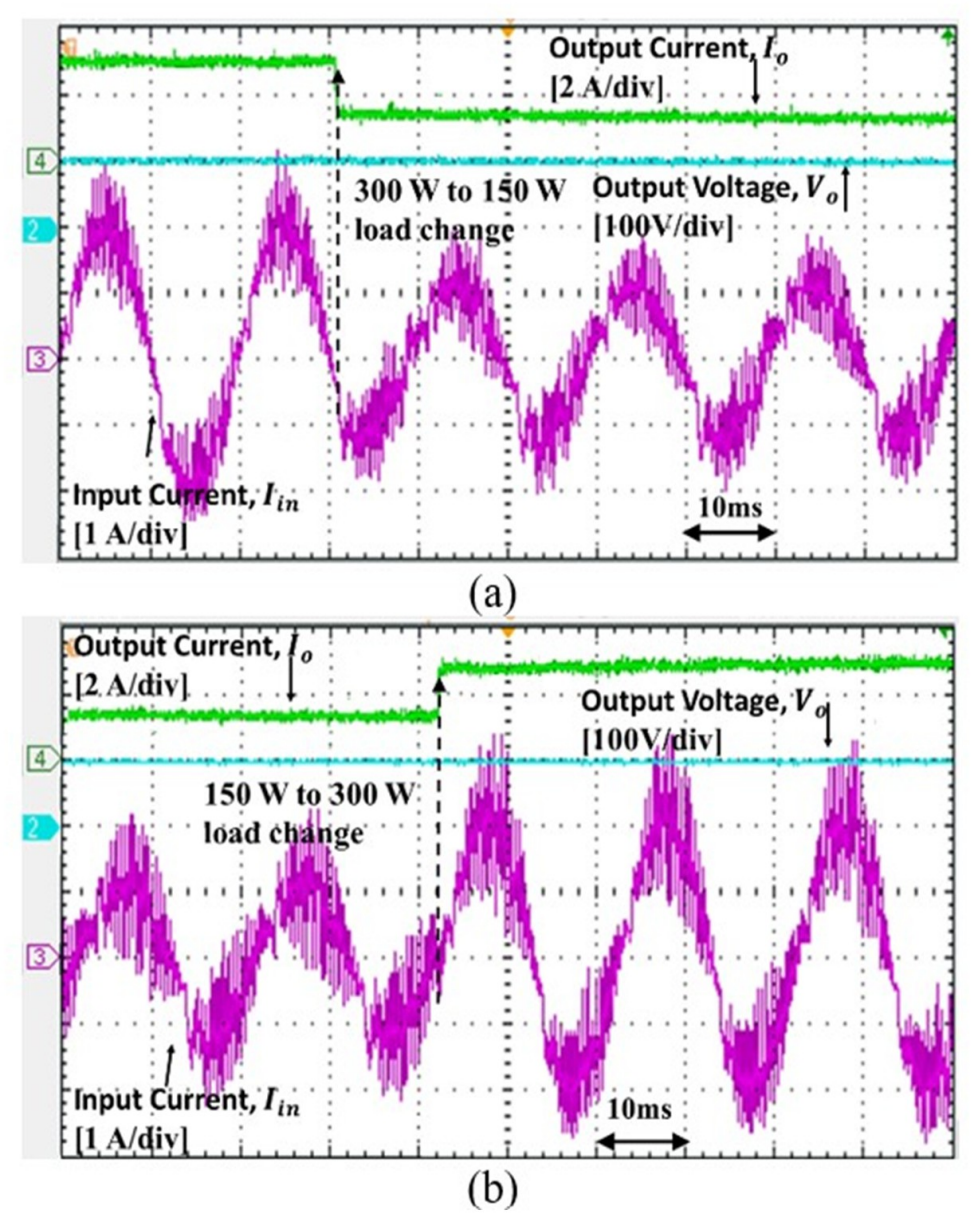
Converter response to the load change for 220 *V*_*in*_*rms* (a) from 300 W to 150 W (b) from 150 W to 300 W.

The experimental results support the simulation results whereby the proposed controller able to reshape the input current sinusoidally. To validate the dynamic performance of the controller, the step load change is performed from 150 W to 300W, as shown in Figs 17 and 18. Based on the results, as the step load change, the input current changing but the output voltage is steadily regulated at 100 V.

**Fig 19:**
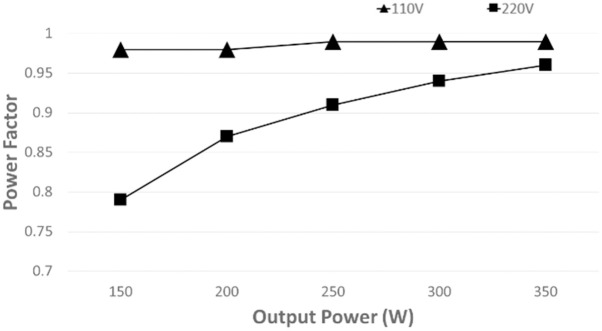
Measured power factor at different power levels of the proposed controller.

Fluke 1730 energy logger is used to measure the power factor, total harmonic distortion (THD), input power, and output power of the converter. The power factor is always greater than 0.78 at all load conditions, as shown in Fig 19. At full load, the power factor accomplishes 0.98 at low input voltage (110 *V*_*in*_*rms*), while at high input voltage (220 *V*_*in*_*rms*), the PF can reach 0.96.

**Fig 20:**
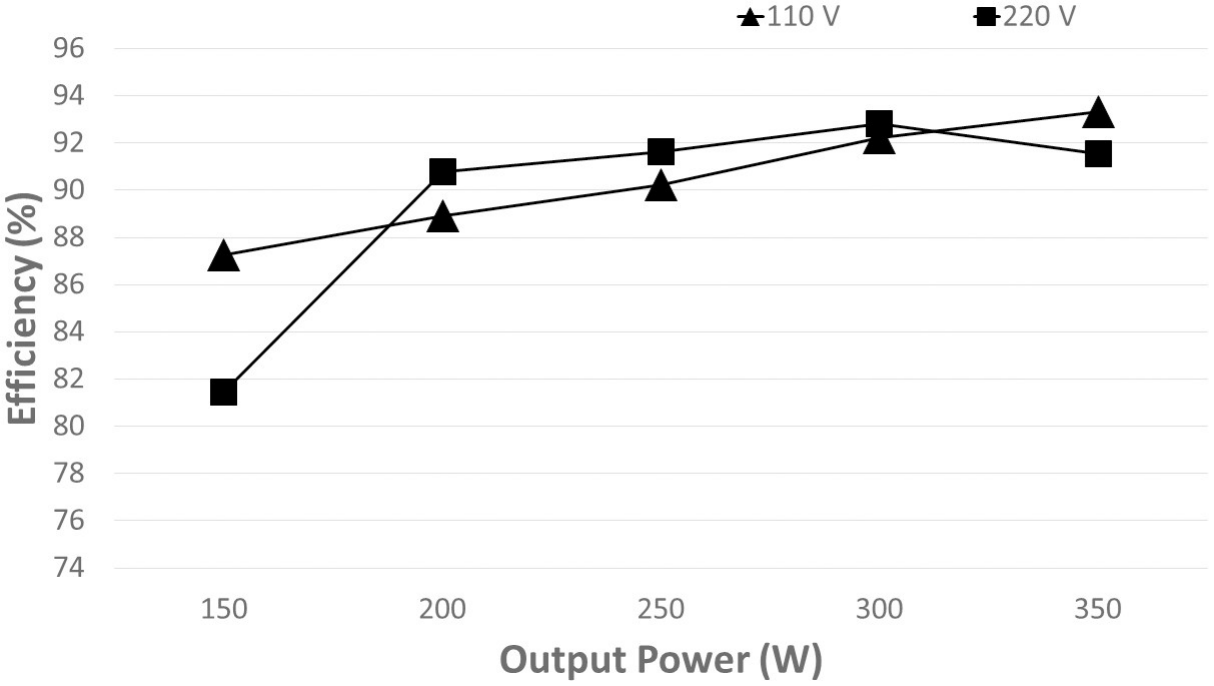
Measured efficiency at different power levels of the proposed controller.

The efficiency of the converter is measured and plotted in Fig 20. Even though the input filter is used at the converter’s front end, the converter’s efficiency still exceeds 78% for all load conditions. As the power increased, the proposed controller attained high efficiency with 91.4% at light load for 110 *V*_*in*_*rms*s.

**Fig 21:**
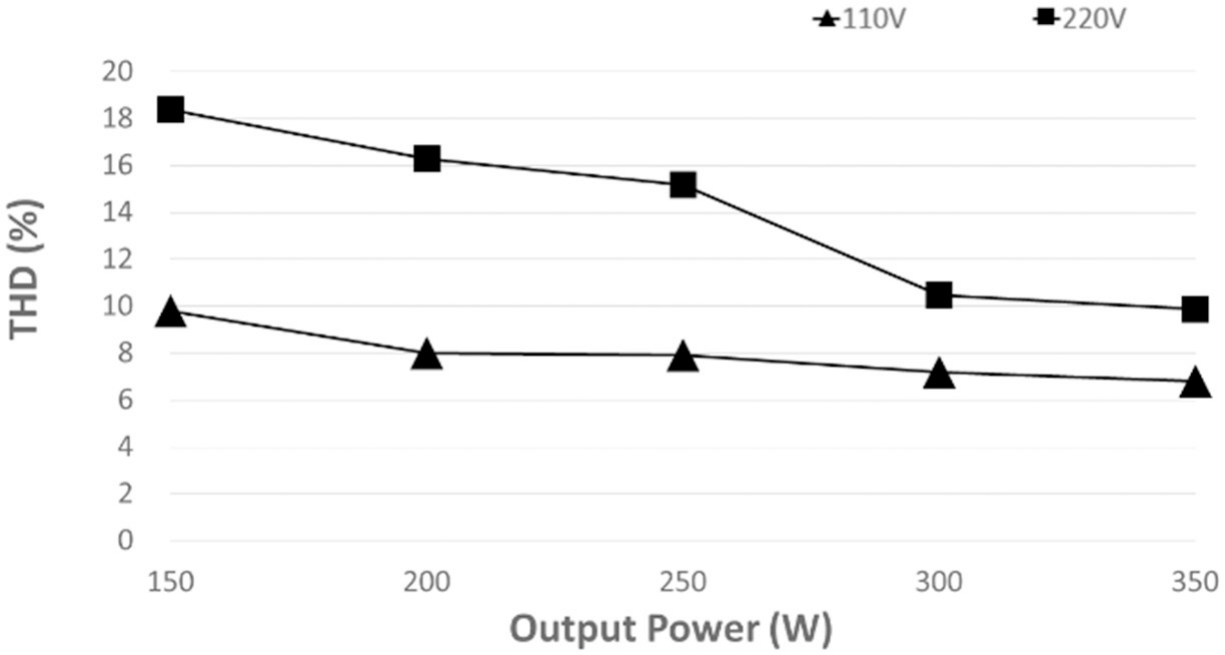
Measured total harmonic distortion (THD) of input current at different power levels of the proposed controller.

**Fig 22:**
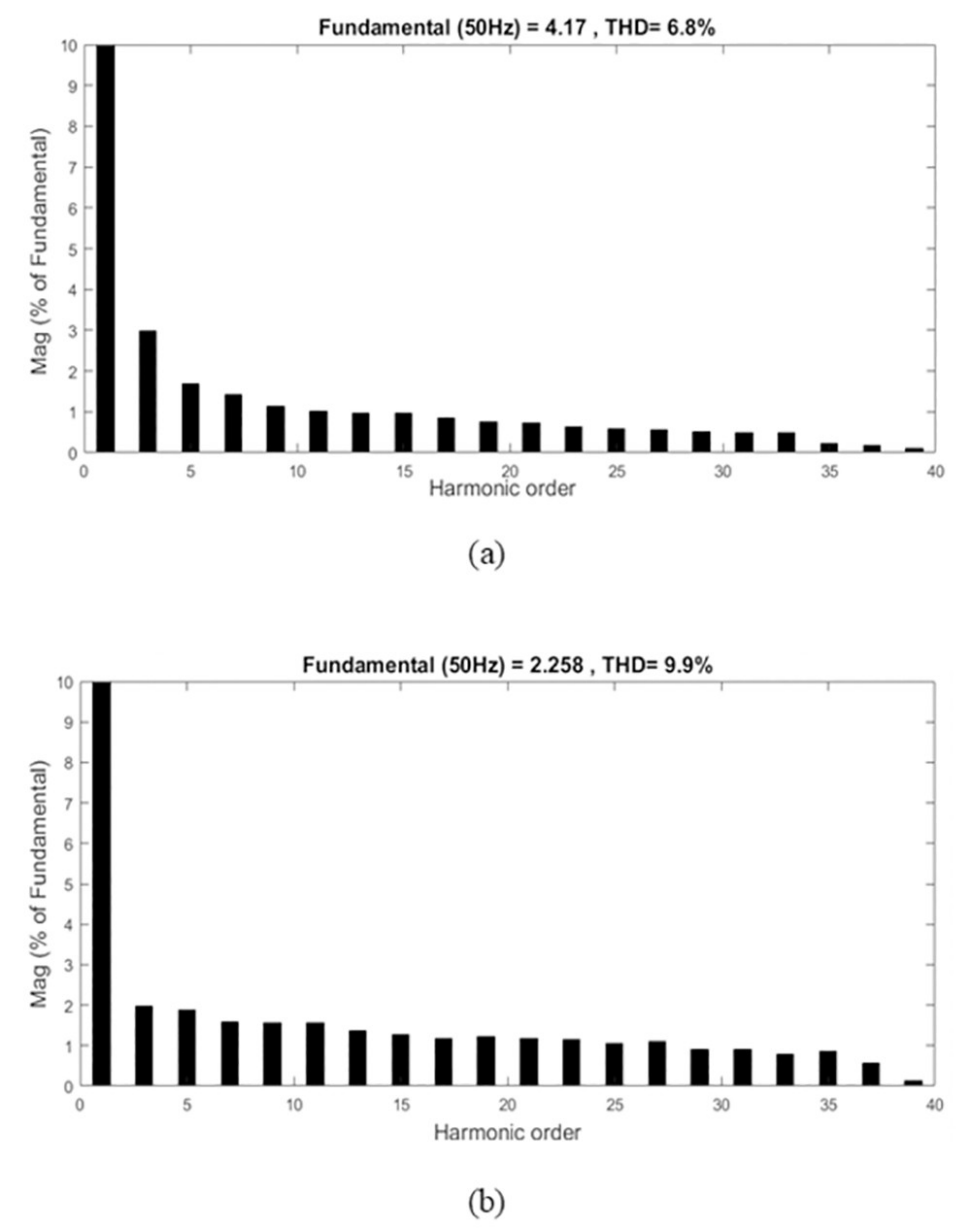
Input current FFT at rated power for (a) 110 V input voltage and (b) 220 V input voltage.

Fig 21 compares the input current THD at a different power level. With the proposed controller, the input current THD significantly improves as the power level increases for both low input voltage and high input voltage. The current input THD measured 6.8% for low input voltage while 9.9% for high input voltage at full load as shown in the fast Fourier transform (FFT) of the input current in Fig 22. It is observed that at medium power, the proposed controller demonstrates that the input current quality is slightly enhanced.

The experiment results have shown that the bridgeless SEPIC performs better at low input voltage, 110 V than at high input voltage, 220 V because it provides low ripple sinusoidal input current, high efficiency, a power factor close to unity, and low THD at all power loads.

## Conclusion

This study aims to implement a single-phase PFC bridgeless SEPIC converter operated in CCM using ACMC via second-order reduction. The proposed controller has been analysed theoretically, proven by simulation, and validated through an experimental test rig. The investigation has concluded that the proposed controller with reduction order model shows a good agreement working in CCM operation using ACMC in both simulation and experimental test-rig. The reduction order model ensures simplicity in the controller’s design without affecting the original system’s dynamic response. The observations from this study suggest that a high power factor greater than 0.98 is the archive for all load conditions for low input voltage.

Meanwhile, as the power increased for the high input voltage side, the power factor improved to 0.96. The input current is perfectly reshaped sinusoidally. The converter’s efficiency is 91.46% for low input voltage and 89.65% for high input voltage at light load even though the input filter is used at the front end of the converter. The controller performs steadily at load changing. It can regulate and reshape input current while maintaining PFC at CCM operation at greater than 150 W power. These findings proved that the bridgeless SEPIC converter could operate at a medium power application with 350 W in CCM operation.
